# Phylogenetic relationships and characterization of the complete chloroplast genome of *Paphiopedilum* ‘GZSLKY Youyou’, a hybrid of *P. dianthum* × *P. barbigerum*

**DOI:** 10.1080/23802359.2022.2070041

**Published:** 2022-05-04

**Authors:** Hengzhao Liu, Hang Ye, Peng Zhao

**Affiliations:** Key Laboratory of Resource Biology and Biotechnology in Western China, Ministry of Education, College of Life Sciences, Northwest University, Xi’an, China

**Keywords:** *Paphiopedilum*, ‘GZSLKY Youyou’, complete chloroplast genome, phylogenetic tree

## Abstract

*Paphiopedilum* ‘GZSLKY Youyou’. is a new cultivar of *Paphiopedilum* with highly ornamental and horticultural value developed by crossing female parent *Paphiopedilum dianthum* T. Tang & F. T. Wang 1940 and male parent *Paphiopedilum barbigerum* T. Tang & F. T. Wang 1940. In this study, the complete chloroplast genome of the cultivar has been reconstructed from the Illumina sequencing data. The circular genome was 160,503 bp in size, containing a large single-copy region (91,582 bp), a small single-copy region (3,215 bp) and a pair of IR regions (each one 32,853 bp). The overall GC contents of the chloroplast genome was 36.20%. The chloroplast genome contained 122 genes, including 76 protein coding genes, 38 tRNA genes and 8 rRNA genes. A maximum likelihood (ML) phylogenetic tree showed that the cultivar ‘GZSLKY Youyou’ was clustered into the same clade with its parents and was closest related to *P. dianthum*, reflecting a maternal inheritance of chloroplasts. This complete chloroplast genome resource could be further used for genomic studies, phylogenetic analyses, and genetic engineering and breeding of the genus *Paphiopedilum*.

The genus *Paphiopedilum*, belonging to Orchidaceae, includes over 60 species of terrestrial, lithophytic, and epiphytic orchids from Asiatic tropics to pacific regions with highly ornamental and horticultural value. However, owing to the destructive exploitation and habitat deterioration of wild germplasm resources of *Paphiopedilum*, it has been listed in Convention on International Trade in Endangered Species of Wild Fauna and Flora (CITES) and prohibited from ruthless collection and international trading (Luo et al. 2003). Under such a circumstance, new cultivars with distinct floral traits of *Paphiopedilum* have been produced by using classical and mutational-breeding tools. And an alternative approach to preserve the genetic pool of an endangered species is to generate hybrids, which can subsequently be selected based on their specific traits (Choi et al. 2020).

Hybridization of *Paphiopedilum* has been performed for more than 150 years. *Paphiopedilum* ‘GZSLKY Youyou’ F. X. Yan et al. 2017 is a new cultivar of *Paphiopedilum* developed by crossing female parent *Paphiopedilum dianthum* T. Tang & F. T. Wang 1940 and male parent *Paphiopedilum barbigerum* T. Tang & F. T. Wang 1940. This cultivar is characterized by the shape and color traits of flowers between the two parents. With green leaves and more flowers, this hybrid cultivar has a high seed germination rate and wide adaptability. It is also characterized by erect stalks, more elegant flowers than its parents, and high flowering efficiency (Yan et al. 2017). Previous researches indicated that the phylogenetic relationships among the genus *Paphiopedilum* were not well clarified based on sparse taxon sampling (Guo et al. 2021). Recently, the chloroplast genome sequence being a dependable tool for phylogenetic and genetic studies, which has been reported in many valuable plants (Chen et al. 2019). To promote its genetic research and resource utilization, for this study, we assembled the complete chloroplast genome of *P.* ‘GZSLKY Youyou’ based on Illumina sequencing platform followed by the phylogenetic analysis, which will be beneficial for comprehensive understanding of the evolution within the Orchidaceae and further investigations on its chloroplast genetic engineering.

The voucher specimen of the hybrid cultivar was stored at the herbarium of Northwest University (108°55′E, 34°15′N, accession number: SK2021206; College of Life Sciences, Northwest University, Hengzhao Liu and hengzhaoliu@stumail.nwu.edu.cn). The total genomic DNA was extracted from fresh leaves using a modified CTAB method (Doyle and Doyle 1987). The libraries of insert sizes of ∼350 bp were constructed from randomly fragmented genomic DNA which broken by the Covaris ultra-sonic shearer. A Truseq Library Prep Kit (Illumina) was used to build the library in the following steps according to the Illumina’s standard protocol: end repair, polyA tail addition, sequencing connector addition, purification, PCR amplification. And then 150 bp paired-end reads were produced using the Illumina HiSeq 2500 sequencing platform. The GetOrganelle (Jin et al. 2020) software was adopted to assemble the pruned reads by using the *P. dianthum* chloroplast genome sequence as a reference (GenBank accession number: NC_036958) (Hou et al. 2018), after which the assembled chloroplast genome was annotated using the online tools GeSeq (Tillich et al. 2017). The accurate new annotated complete chloroplast genome was submitted to GenBank with the accession number MN315105.

The chloroplast genome of *P.* ‘GZSLKY Youyou’ was a double-stranded circular DNA molecule with 160,503 bp in size. It comprised a pair of inverted repeat (IR) regions of 32,853 bp each, separated by a large single-copy (LSC) region of 91,582 bp and a small single-copy (SSC) region of 3,215 bp. The total GC content is 36.20%, while the corresponding values of the IR, LSC, and SSC region are 20.5%, 57.1% and 2%, respectively. This chloroplast genome comprised 122 functional genes, including 76 protein-coding genes (PCGs), 38 tRNA genes and 8 rRNA genes.

To conduct phylogenetic analysis, we downloaded complete chloroplast genome sequences of 16 other Orchidaceae species from NCBI and two Liliaceae species as outgroup. The sequence alignment was implemented using MAFFT software (Katoh and Standley 2013). After purning by GBLOCKS, the phylogenetic tree was constructed based on maximum-likelihood (ML) method using the IQ-TREE program (Minh et al. 2020) under the best-fit model GTR + F+R3 with 1,000 times ultrafast bootstrap replicates. The resulting of phylogeny showed that hybrid *P.* ‘GZSLKY Youyou’ was clustered in the genus *Paphiopedilum* and was most closely related to *P. dianthum* with 100% bootstrap, which reflects a maternal inheritance of chloroplast genome (Figure 1).

**Figure 1.**
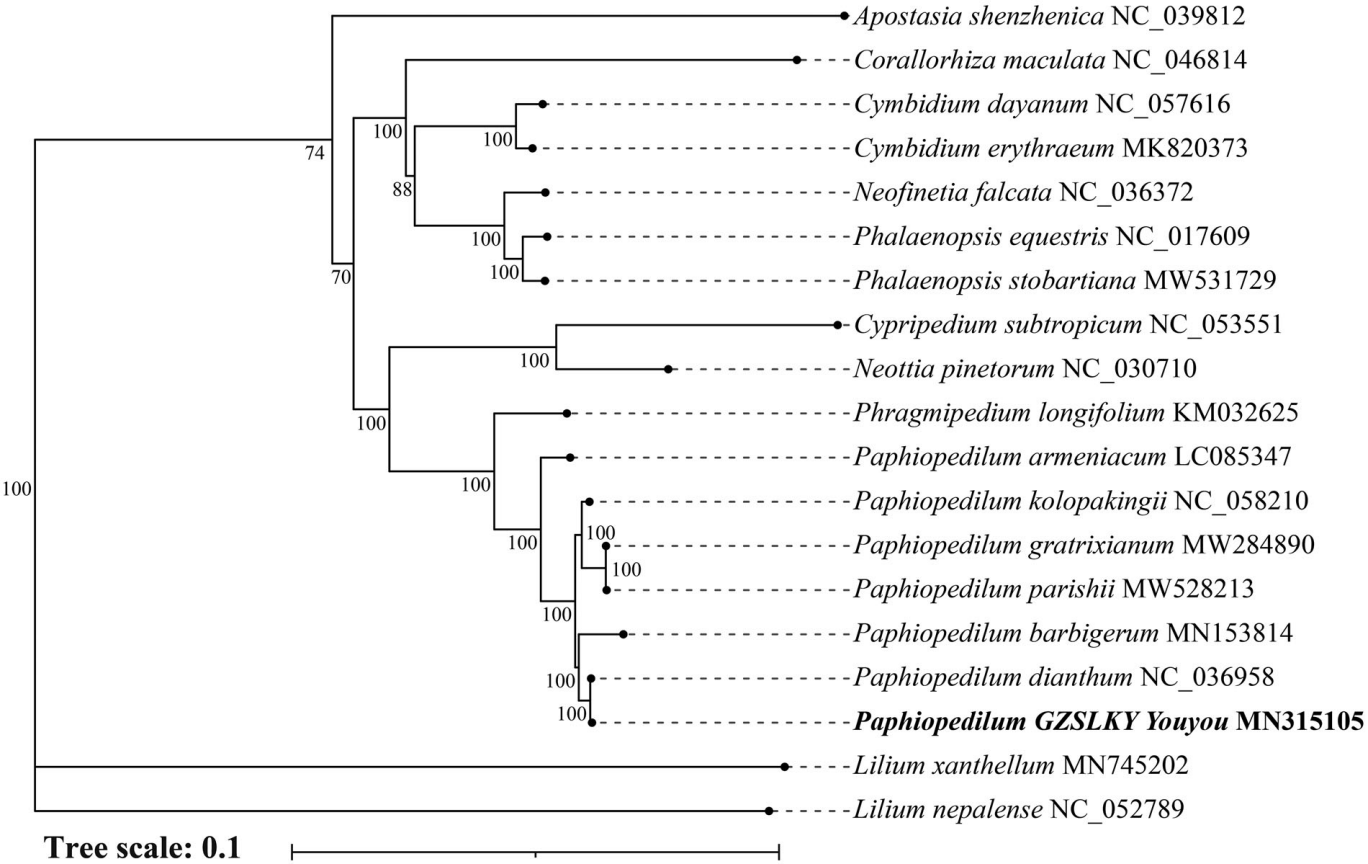
Maximum likelihood (ML) phylogenetic tree inferred from chloroplast genome sequences of 17 orchid species and 2 outgroups. Accession numbers were listed behind each taxon. Numbers on nodes indicated bootstrap values.

Owning to the horticultural value and extinction crisis, as the most primitive and largest genus of Orchidaceae, *Paphiopedilum* has been attracting attention by biologists continuously for utilization and conservation. Additionally, *Paphiopedilum* provides a unique opportunity to study the dynamics of the boundary shift impact on plastid genome structure and sequence evolution, and many deep-level relationships of the genus remain unclear as yet (Kim et al. 2015; Guo et al. 2021). Overall, as an important genetic and molecule resource, the publication of the chloroplast genome of the hybrid would not only supply a valuable foundation for the development and conservation of the plant germplasm, but also provide a new opportunity and perspective for the research on the evolution, comparative genomics and genetic improvement of *Paphiopedilum*.

## Data Availability

The complete chloroplast genome sequence data that support the findings of this study was submitted to GenBank under the accession number of MN315105 (https://www.ncbi.nlm.nih.gov/nuccore/MN315105.1/). The associated BioProject, SRA, and Bio-Sample numbers are PRJNA791767, SRR17318925, and SAMN24344705, respectively.

## References

[CIT0001] Chen L, Li L, Yang G, Qian H, Li M. 2019. Characterization of the complete chloroplast genome sequence of *Tsuga longibracteata* W. C. Cheng (Pinaceae). Conservation Genet Resour. 11(2):117–120.

[CIT0002] Choi HI, Lyu JI, Lee HO, Kim JB, Kim SH. 2020. Complete chloroplast genome sequence of an orchid hybrid *Cymbidium sinense* (**♀**) x C. goeringii (**♂**). Mitochondrial DNA B. 5:3802–3803. 10.1080/23802359.2020.1839367 PMC7682733 33367106

[CIT0003] Doyle JJ, Doyle JL. 1987. A rapid DNA isolation procedure for small quantities of fresh leaf tissue. Phytochem Bull. 19:11–15.

[CIT0004] Guo YY, Yang JX, Bai MZ, Zhang GQ, Liu ZJ. 2021. The chloroplast genome evolution of Venus slipper (Paphiopedilum): IR expansion, SSC contraction, and highly rearranged SSC regions. BMC Plant Biol. 21(1):248. 34058997 10.1186/s12870-021-03053-y PMC8165784

[CIT0005] Hou N, Wang G, Zhu Y, Wang L, Xu J. 2018. The complete chloroplast genome of the rare and endangered herb *Paphiopedilum dianthum* (Asparagales: Orchidaceae). Conservation Genet Resour. 10(4):709–712.

[CIT0006] Jin JJ, Yu WB, Yang JB, Song Y, dePamphilis CW, Yi TS, Li DZ. 2020. GetOrganelle: a fast and versatile toolkit for accurate de novo assembly of organelle genomes. Genome Biol. 21(1):241. 32912315 10.1186/s13059-020-02154-5 PMC7488116

[CIT0007] Katoh K, Standley DM. 2013. MAFFT multiple sequence alignment software version 7: improvements in performance and usability. Mol Biol Evol. 30(4):772–780. 23329690 10.1093/molbev/mst010 PMC3603318

[CIT0008] Kim HT, Kim JS, Moore MJ, Neubig KM, Williams NH, Whitten WM, Kim JH. 2015. Seven new complete Plastome sequences reveal rampant independent loss of the Ndh gene family across orchids and associated instability of the inverted repeat/small single-copy region boundaries. PLoS One. 10(11):e0142215. 26558895 10.1371/journal.pone.0142215 PMC4641739

[CIT0009] Luo Y, Jia J, Wang C. 2003. Conservation strategy and potential advantages of the Chinese *Paphiopedilum*. Biodiv Sci. 11:491–498.

[CIT0010] Minh BQ, Schmidt HA, Chernomor O, Schrempf D, Woodhams MD, von Haeseler A, Lanfear R. 2020. IQ-TREE 2: new models and efficient methods for phylogenetic inference in the genomic era. Mol Biol Evol. 37(5):1530–1534. 32011700 10.1093/molbev/msaa015 PMC7182206

[CIT0011] Tillich M, Lehwark P, Pellizzer T, Ulbricht-Jones ES, Fischer A, Bock R, Greiner S. 2017. GeSeq - versatile and accurate annotation of organelle genomes. Nucleic Acids Res. 45(W1):W6–W11. 28486635 10.1093/nar/gkx391 PMC5570176

[CIT0012] Yan F, Tian F, Jiang Y, Luo Z, Wang P. 2017. A New *Paphiopedilum* Cultivar‘GZSLKY Youyou. Acta Horticulturae Sinica. 44:1221–1222.

